# Transcriptional Activator GmrA, Encoded in Genomic Island OI-29, Controls the Motility of Enterohemorrhagic *Escherichia coli* O157:H7

**DOI:** 10.3389/fmicb.2018.00338

**Published:** 2018-02-22

**Authors:** Bin Yang, Shaomeng Wang, Jianxiao Huang, Zhiqiu Yin, Lingyan Jiang, Wenqi Hou, Xiaomin Li, Lu Feng

**Affiliations:** ^1^TEDA Institute of Biological Sciences and Biotechnology, Nankai University, Tianjin, China; ^2^Key Laboratory of Molecular Microbiology and Technology, Ministry of Education, Tianjin, China; ^3^Tianjin Key Laboratory of Microbial Functional Genomics, Tianjin, China; ^4^State Key Laboratory of Medicinal Chemical Biology, Nankai University, Tianjin, China; ^5^SynBio Research Platform, Collaborative Innovation Center of Chemical Science and Engineering (Tianjin), Tianjin, China

**Keywords:** enterohemorrhagic *Escherichia coli*, O island 29, Z0639, motility, flagellar synthesis

## Abstract

Enterohemorrhagic *Escherichia coli* O157:H7 is a major human enteric pathogen capable of causing large outbreaks of severe infections that induce bloody diarrhea, hemorrhagic colitis, and hemolytic uremic syndrome. Its genome contains 177 unique O islands (OIs) including those carrying the main virulence elements, Shiga toxin-converting phages (OI-45 and OI-93) and locus for enterocyte effacement (OI-148). However, many of these islands harbor only genes of unknown function. Here, we demonstrate that OI-29 encodes a newly discovered transcriptional activator, Z0639 (named GmrA), that is required for motility and flagellar synthesis in O157:H7. GmrA directly binds to the promoter of *fliA*, an RNA polymerase sigma factor, and thereby regulates flagellar genes controlled by FliA. Expression of *gmrA* is maximal under host conditions (37°C, neutral pH, and physiological osmolarity), and in the presence of host epithelial cells, indicative of a role of this gene in infection by promoting motility. Finally, GmrA was found to be a widespread regulator of bacterial motility and flagellar synthesis in different pathotypes of *E. coli*. Our work largely enriches our understanding of bacterial motility control, and provides another example of regulators acquired laterally that mediate flagellar synthesis.

## Introduction

Enterohemorrhagic *Escherichia coli* (EHEC) is a principally foodborne pathogen linked to serious diseases, including hemorrhagic colitis and hemolytic uremic syndrome (Monteiro et al., 2016). Adult cattle and other farm animals are the main reservoirs of many EHEC serotypes (Caprioli et al., 2005), and humans become infected via contaminated food and water (Watanabe, 2012; Monteiro et al., 2016). The major virulence determinants of EHEC include the ability to produce Shiga toxins, adhere to host epithelial cells, and form histopathological attaching and effacing lesions (Watanabe, 2012). Of which, the induction of such lesions is most critical for the establishment of successful colonization to cause infection, and this ability is conferred by locus of enterocyte effacement (LEE), which consists of five polycistronic operons (LEE1 to LEE5) encoding a type III secretion system and associated effectors (Wong et al., 2011; Monteiro et al., 2016). Gene expression from this locus is regulated via a complex mechanism to ensure the expression occurs only under host conditions (Mellies et al., 2007; Connolly et al., 2015). While LEE1-encoded *ler* is the master activator of all LEE operons, a range of global and specific regulators are also involved, such as H-NS, IHF, QseA, GrvA, GadE, Pch, EivF, EtrA, and Hha (Connolly et al., 2015).

Bacterial motility is often considered to play diverse roles in pathogenesis, including in migration to an optimal site in the host, colonization or invasion, survival at the infection site, and post-infection dispersal (Chaban et al., 2015). One of the most widespread motility machines in bacteria is the flagellum, a complex macromolecular structure driven by a motor which rotates a long, curved filament extending from the cell envelope (Berg, 2003; Chaban et al., 2015). A flagellum consists of a basal body (rotary motor), a hook (universal joint), and a filament (propeller) formed through polymerization of flagellin (FliC) (Erhardt et al., 2010). More than 50 genes are required to form and operate the flagellum, the expression of which is stringently controlled to minimize unnecessary energy expenditure. The regulation of flagellar gene expression is highly complex, with FlhD and FlhC as master transcriptional activators (Claret and Hughes, 2000). These proteins form an FlhD_4_C_2_ complex and boost expression of flagellar genes, both directly and via FliA, an RNA polymerase sigma factor (Claret and Hughes, 2000; Chevance and Hughes, 2008). Flagellar synthesis is also regulated by various proteins and sRNAs, including MatA, CRP, H-NS, HdfR, QseBC, and DksA, which regulate *flhDC* transcriptionally, and thereby control *fliA* and other flagellar genes (McCarter, 2006; Duan et al., 2013), and H-NS, CsgD, and NsrR, that control flagella-based motility by modulating *fliA* transcription (McCarter, 2006; Duan et al., 2013).

*Escherichia coli* O157:H7, the most well-known EHEC strain, is also the most common serotype associated with large infection outbreaks (Bavaro, 2012; Ho et al., 2013). Notably, the O157:H7 genome contains 177 O islands (OI) that are not present in non-pathogenic *E. coli* K-12 (Perna et al., 2001). These islands comprise the main known virulence elements in O157:H7, and include LEE (OI-148) and Shiga toxin-converting phages (OI-45 and OI-93) (Hayashi et al., 2001; Perna et al., 2001). The functions of several other islands were also established in recent years, revealing more virulence factors associated with adherence and motility. OI-1 was found to encode a repressor of flagellar synthesis and bacterial motility, while OI-172 was determined to encode an activator (Allison et al., 2012; Xu et al., 2013). OI-15 is now known to encode an AIDA-like adhesin required for adherence *in vitro* and *in vivo* (Yin et al., 2009a), while OI-48 was determined to encode tellurite resistance, Iha, and urease to promote adherence to the host intestinal epithelium (Yin et al., 2009b). OI-50 and OI-51 encode virulence regulators and other effectors required for infection (Tree et al., 2011; Flockhart et al., 2012), while OI-71 encodes NleA, a type III secretion system effector encoded outside of LEE (Gruenheid et al., 2004). OI-122 also carries the virulence genes *efa1/lifA*, which encode adherence/lymphocyte inhibitory factor and is required for pathogen adhesion *in vitro* and suppression of the host immune response (Karmali et al., 2003). Nevertheless, most genes in O islands have not been characterized and are of unknown function.

We now demonstrate that Z0639, renamed as GmrA (Genomic island-encoded Motility Regulator A), encoded in OI-29 is a newly discovered transcriptional activator that regulates flagellar synthesis and motility in *E. coli* O157:H7. GmrA directly binds to the promoter of *fliA* based on EMSA and ChIP-qPCR analysis, and thereby regulates flagellar genes controlled by FliA. *gmrA* expression is maximal at 37°C, neutral pH, physiological osmolarity, and in the presence of host epithelial cells. Finally, GmrA was found to be a widespread regulator of bacterial motility in pathogenic *E. coli*. This work reveals a new example of regulators acquired laterally for the control of flagella synthesis.

## Materials and Methods

### Bacterial Strains, Plasmids, and Culture

Bacterial strains and plasmids are summarized in Supplementary Table S1. Mutant strains were generated by the Red recombinase system (Datsenko and Wanner, 2000; Murphy and Campellone, 2003), and verified by PCR and sequencing. Complemented strains were constructed by cloning the appropriate genes into low-copy plasmid pACYC184, and then by electroporating the resulting constructs into the corresponding mutants. Strains for protein purification were constructed by cloning genes of interest into the pET28a expression vector, and then by electroporating the resulting constructs into *E. coli* BL21. All genetic manipulation on virulent bacterial strains was performed according to standard biosecurity and institutional safety procedures. Primers for all manipulations are listed in Supplementary Table S2. Unless otherwise specified, all strains were grown in Luria-Bertani (LB) broth supplemented as needed with 100 μg mL^-1^ ampicillin, 15 μg mL^-1^ chloramphenicol, and 50 μg mL^-1^ kanamycin.

### Bacterial Adherence

Adherence was assayed as previously described (Dibb-Fuller et al., 2001). Briefly, HeLa and Caco-2 cells, obtained from Shanghai Institute of Biochemistry and Cell Biology, Chinese Academy of Sciences (Shanghai, China), were grown at 37°C in 5% CO_2_ until confluent, washed three times with pre-warmed PBS, and the medium was replaced with fresh DMEM without antibiotics and fetal bovine serum. Cells were then infected with bacterial cultures in exponential phase (10^8^ CFU/well). After 3 h, unattached bacteria were removed by washing the wells six times with PBS. Cells were then lysed with 0.1% SDS, and resulting lysates were serially diluted and plated on LB agar. Attachment efficiency was calculated as the numbers of adherent bacteria per cell.

### Quantitative Real-Time PCR (qRT-PCR)

Total RNA was prepared using TRIzol^®^ LS Reagent (Invitrogen: 15596018) following the manufacturer’s instructions, and digested with RNase-Free DNase I (Qiagen: 79254) to eliminate contaminating genomic DNA. First-strand cDNA was synthesized using PrimeScript 1^st^ Strand cDNA Synthesis Kit (Takara: D6110A), and analyzed by qRT-PCR on an ABI 7500 system (Applied Biosystems) using SYBR Green PCR master mix (Applied Biosystems: 4367659). The 16S rRNA gene *rrsH* was used as reference, and relative differences in gene expression were calculated by the cycle threshold method (2^-ΔΔct^) (Livak and Schmittgen, 2001; Tasara and Stephan, 2007). Data were collected from at least three biological replicates.

### Motility

Overnight cultures were adjusted to optical density 1.0 at 600 nm, of which 1 μL was then stab-inoculated using a sterile pipette tip into 0.25% LB-agar plates supplemented with ampicillin as needed. Agar plates were then incubated at 30 or 37°C for 10 h, at which point the diameter of the swimming zone around the inoculation site was measured. All strains were tested in triplicate, and each experiment was carried out on three separate occasions.

### Transmission Electron Microscopy

Strains were cultured in LB broth at 37°C until optical density 0.8 at 600 nm. Samples (1 mL) were then harvested at 2,000 rpm for 5 min, resuspended in an equal volume of distilled water, of which 10 μL was dropped and adsorbed for 3 min to carbon-stabilized Formvar supports on 200-mesh copper grids. Cells were then stained by submerging the grids for 3 min in 2% wt/vol sodium phosphotungstate, and imaged on a HITACHI HT7700 transmission electron microscope operating at 100 kV and fitted with a high-sensitivity real-time CCD camera.

### Western Blotting

Overnight bacterial cultures were diluted 1:100 to optical density 1.0 at 600 nm. Whole-cell lysates were resolved on 12% sodium dodecyl sulfate-polyacrylamide gels, and transferred to polyvinylidene difluoride membranes. Subsequently, membranes were probed with a 1:10,000 dilution of antibodies to flagellin (Abcam: 93713) or DnaK (Abcam: ab69617), followed by a 1:2,000 dilution of goat anti-rabbit (Abcam: ab6721) or anti-mouse immunoglobulin G (Abcam: ab205719) conjugated to horseradish peroxidase. Blots were visualized on a chemiluminescence detection system following reaction with ECL enhanced chemiluminescence reagent. Proteins were quantified using Amersham Imager 600 software (GE Healthcare).

### Electrophoretic Mobility Shift Assay

GmrA N-terminally tagged with 6× His was expressed in *E. coli* BL21, using the expression vector pET28a and purified from soluble extracts using nickel columns (GE Healthcare: 17057501). Protein concentration was determined by Bradford assay, and stored in aliquots at -70°C. PCR fragments encompassing regulatory regions of *fliA* (495 bp, -412 to +83) and *flhD* (541 bp, -336 to +205), with respect to the corresponding transcriptional start sites, were amplified using genomic DNA of *E. coli* O157:H7 EDL933 as template (for the regulatory regions of *fliA* and *flhD*, see Salgado et al., 2013). A *rpoS* fragment (384 bp, +1052 to +1435 relative to the transcriptional start site) was also amplified, and used as negative control. The DNA fragments were then gel-purified and labeled with DIG using terminal transferase. Eectrophoretic mobility shift assays were performed using DIG Gel Shift Kit, 2^nd^ Generation (Roche: 03353591910) according to the manufacturer’s instructions. Briefly, labeled DNA fragments (1 nM) were incubated at 37°C for 20 min with various concentrations of purified GmrA -His6 (0–120 nM), in 20 μL reactions containing band-shift buffer (20 mM Tris-HCl pH 7.5, 80 mM NaCl, 0.1 mM EDTA, and 1 mM DTT). For competition assays, various concentrations of unlabeled DNA fragments (10–150 nM) were added. Samples were separated by 10% native polyacrylamide gel electrophoresis, and transferred to nylon membranes. Labeled fragments were visualized on a chemiluminescence detection system following an enzyme immunoassay using anti-digoxigenin-AP, Fab-fragments, and the chemiluminescent substrate CSPD.

### Chromatin Immunoprecipitation-Quantitative PCR (ChIP-qPCR)

Chromatin immunoprecipitation was performed as previously described (Lucchini et al., 2006; Davies et al., 2011) with some modification. Briefly, an inducible expression vector (pTRC99a) carrying 3× FLAG-tagged *gmrA* was constructed and transformed into Δ*gmrA* mutant. Bacterial cultures were grown to mid-logarithmic phase until optical density 0.4 at 600 nm, and protein expression was induced with 1 mM IPTG for 30 min at 37°C. To crosslink protein to DNA, formaldehyde was added to cultures to a final concentration of 1%, and the mixture incubated at room temperature for 25 min. Cross-linking was quenched by adding glycine at a final concentrations of 0.5 M. Cross-linked cells were then washed three times with ice-cold TBS, and sonicated extensively to generate DNA fragments of average size ∼500 bp. Cell debris was removed, and the resulting supernatant was used as cell extract for immunoprecipitation. Protein-DNA complexes were enriched with 3× FLAG antibody (Sigma: F1804) and protein A magnetic beads (Invitrogen: 10002D), following the manufacturer’s instructions. As negative control, chromatin immunoprecipitation was performed using different aliquot without addition of antibodies. RNA were removed by incubation with RNaseA for 2 h at 37°C, and proteins were removed by incubation with proteinase K for 2 h at 55°C. The DNA sample was then purified using a PCR purification kit (Qiagen: 28104). To measure enrichment of *fliA* and *flhDC* promoters in immunoprecipitated DNA samples, relative-abundance quantitative PCR (qPCR) was performed with SYBR green mix. Relative enrichment was calculated by the ΔΔCt method (Livak and Schmittgen, 2001). Results shown represent average enrichment for three biological replicates.

### Bioinformatics

Orthologous groups were identified using OrthoFinder (Emms and Kelly, 2015), by which all nucleotide sequences were compared using a BLASTN all-against-all search with an E-value cutoff of <10^-4^. Nucleotide sequences used to construct the phylogenetic tree were aligned in MAFFT (Katoh and Standley, 2013), and a maximum likelihood tree was constructed in PhyML (Guindon and Gascuel, 2003) based on the GTR model of nucleotide substitution with c-distributed rates among sites.

## Results

### OI-29 Is Not Required for O157:H7 Adherence and LEE Gene Expression

**FIGURE 1:**
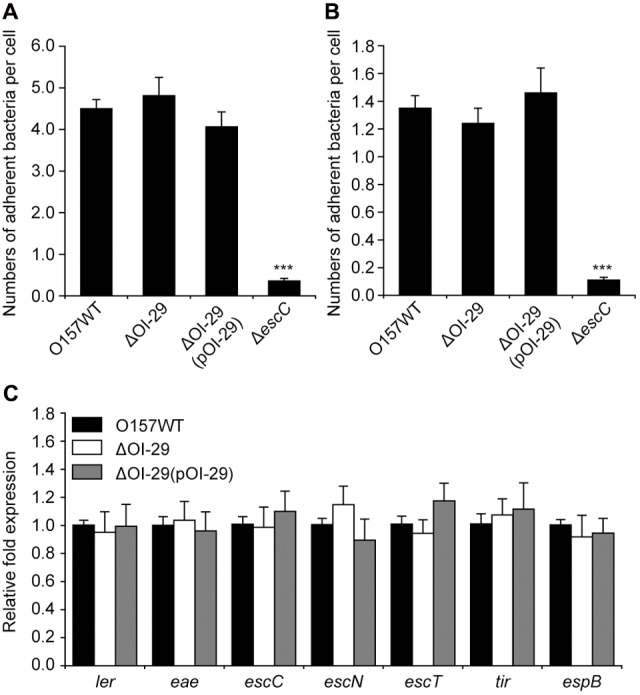
Effect of OI-29 on *Escherichia coli* O157:H7 adherence and LEE gene expression. **(A,B)** Adhered bacteria were quantified 3 h after co-incubating *E. coli* O157:H7 wild-type, ΔOI-29 mutant, ΔOI-29 complemented strain, and Δ*escC* (positive control) with HeLa **(A)** or Caco-2 cells **(B)**. **(C)** *E. coli* strains were also grown to exponential phase, and analyzed by qRT-PCR for the expression of LEE genes, using 16S rRNA as internal control. Data are mean ± SD, *n* = 3. ^∗∗∗^*P* ≤ 0.001 by Student’s *t*-test.

Previously, we showed by comparative transcriptomics that genes in OI-29 are significantly downregulated 3 h after incubation of *E. coli* O157:H7 with HeLa cells (Yang et al., 2015). We have now confirmed this result by qRT-PCR (Supplementary Table S3). To further investigate whether OI-29 is associated with virulence, we constructed a ΔOI-29 mutant and assessed its ability to adhere to host epithelial cells. This mutant was found to similarly adhere to HeLa cells (**Figure 1A**) and Caco-2 intestinal epithelial cells (**Figure 1B**) as the parental strain, while Δ*escC*, a mutant of LEE genes and used as positive control, adhered at much lower levels (**Figures 1A,B**). Accordingly, transcripts of seven representative LEE genes (*ler*, *escT*, *escC*, *escN*, *eae*, *tir*, and *espB*) were similarly abundant between parental and ΔOI-29 strains (**Figure 1C**). These results suggest that OI-29 is not required for *E. coli* O157:H7 adherence and LEE gene expression.

### Deletion of OI-29 Reduces Motility and Flagellar Biosynthesis in O157:H7

**FIGURE 2:**
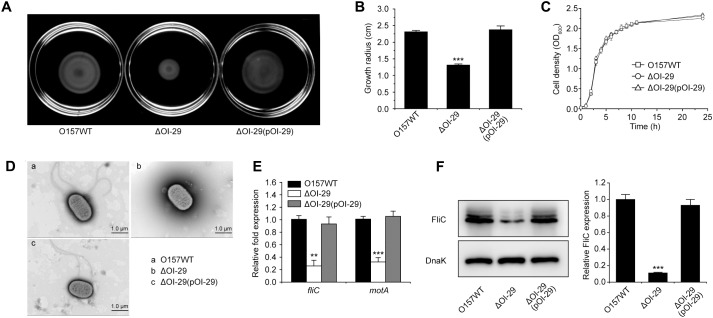
Effect of OI-29 on *E. coli* O157:H7 motility. Representative images of swimming motility **(A)**, growth radius after 10 h at 30°C on motility agar **(B)**, growth in LB medium **(C)**, and representative transmission electron micrographs (**D**; scale bar, 1 μm) of *E. coli* O157:H7 wild-type, ΔOI-29 mutant, and complemented strain. The average number of flagella per cell, as estimated from 500 cells per strain, is 1.21 for wild-type, 0.25 for ΔOI-29 mutant, and 1.37 for the complemented strain. **(E)** Strains grown to exponential phase were analyzed by qRT-PCR for *fliC* and *motA*, using 16S rRNA as internal control. **(F)** Strains were also analyzed by immunoblotting for FliC, using DnaK as loading control. Bands were quantified by densitometry and normalized to DnaK. **(B,E,F)** Data are mean ± SD, *n* = 3. ^∗∗^*P* ≤ 0.01; ^∗∗∗^*P* ≤ 0.001 by Student’s *t*-test.

Motility was repressed in the ΔOI-29 mutant compared with that in wild-type *E. coli* O157:H7, with growth radius after 10 h at 30°C on motility agar 1.86-fold smaller in the former than in the latter (**Figures 2A,B**). The motility defect was rescued by complementation with a low-copy plasmid (pACYC184) encoding OI-29 (**Figures 2A,B**). The motility of ΔOI-29 was not affected by introducing an empty pACYC184 into the mutant (Supplementary Figures S1A,B). The decreased motility of ΔOI-29 was also detected when the bacterium was grown at 37°C (Supplementary Figure S2). Of note, wild-type, ΔOI-29, and the complemented strain grew at similar rates in LB medium (**Figure 2C**), indicating that the decreased motility in ΔOI-29 was not due to slower growth. Strikingly, electron microscopy revealed that approximately 85% of wild-type and complemented cells (*n* = 500 cells per strain) possessed 1–3 flagella, while approximately 80% of ΔOI-29 cells (*n* = 500) were aflagellar. Representative transmission electron micrographs were shown in **Figure 2D**. These results suggested that loss of OI-29 represses flagellar biosynthesis.

Hence, expression of the flagellar genes *fliC* (encoding the major flagellin) and *motA* (encoding the flagella motor) was analyzed by qRT-PCR in both the parental strain and the ΔOI-29 mutant. Deletion of OI-29 was found to decrease the abundance of *fliC* and *motA* transcripts by fourfold and threefold, respectively (**Figure 2E**). The transcription of flagellar genes in ΔOI-29 was not affected by introducing an empty pACYC184 into the mutant (Supplementary Figure S1C). Western blotting also revealed that FliC expression was significantly decreased in the mutant (**Figure 2F**). Transcription and translation were restored to wild-type levels upon complementation with a functional copy of ΔOI-29 (**Figures 2E,F**), confirming that OI-29 enhances *E. coli* O157:H7 motility by upregulating flagellar synthesis.

### GmrA Is an Activator of Motility and Flagellar Biosynthesis in O157:H7

OI-29 in *E. coli* O157:H7 is a 2,643-bp island that contains *z0638*, *z0639* (named *gmrA*), and *z0640*, which encode hypothetical proteins of unknown function (Supplementary Figure S3A). Notably, the *gmrA* gene product contains a putative DNA-binding domain (Supplementary Figure S3B), and therefore may act as a transcriptional regulator. Accordingly, the growth radius of ΔOI-29 on motility plates was restored to wild-type levels when complemented with *gmrA* but not with *z0638* and *z0640* (Supplementary Figure S4), suggesting that the motility defect is due only to *gmrA*. A Δ*gmrA* mutant was also constructed, and was found to have significantly reduced motility on semi-solid LB agar and flagellar biosynthesis, as well as suppressed transcriptional and translational expression of flagellar genes in comparison to wild-type (Supplementary Figures S1D, S5). These defects were comparable to those in ΔOI-29, and were restored to wild-type levels when complemented with a low-copy plasmid carrying *gmrA* (Supplementary Figure S5). On the other hand, the growth radius and transcriptional expression of *fliC* and *motA* were not affected by deletion of *z0638* and *z0640* (Supplementary Figure S6), implying that these genes do not regulate bacterial motility. Collectively, these results confirm that GmrA is an activator of motility and flagellar synthesis in *E. coli* O157:H7.

### GmrA Activates Expression of Flagellar Genes via *fliA*

**FIGURE 3:**
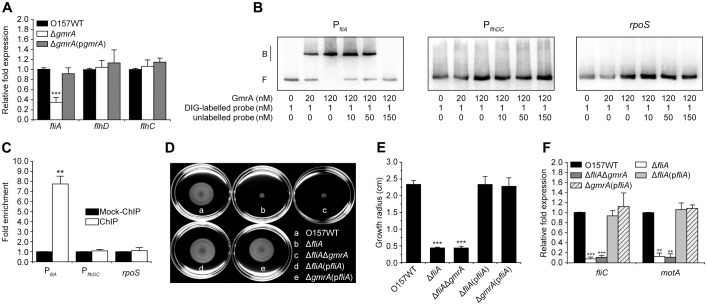
GmrA regulates *E. coli* O157:H7 motility and flagellar synthesis through *fliA*. **(A)** Expression of *fliA*, *flhD*, and *flhC* in *E. coli* O157:H7 wild-type, Δ*gmrA* mutant, and complemented strain, as measured by qRT-PCR of cells in exponential phase, using 16S rRNA as internal control. **(B)** Electrophoretic mobility shift and competition assays of GmrA against the *fliA* and *flhDC* promoters, as well as *rpos* (negative control), with bound and free fragments marked B and F, respectively, and concentrations indicated at the bottom of each lane. Full blots are shown in Supplementary Figure S11. **(C)** Fold enrichment of *fliA* and *flhDC* promoters in GmrA-ChIP samples, as measured by ChIP-qPCR using *rpoS* as negative control. Representative images of swimming motility **(D)** and growth radius after 10 h at 30°C on motility agar **(E)** of *E. coli* O157:H7 wild-type, Δ*fliA* mutant, Δ*fliA*Δ*gmrA* double mutant, and corresponding complemented strains. **(F)** qRT-PCR for *fliC* and *motA* in cells grown to exponential phase, using 16S rRNA gene as internal control. **(A,C,E,F)** Data are mean ± SD, *n* = 3. ^∗∗^*P* ≤ 0.01; ^∗∗∗^*P* ≤ 0.001 by Student’s *t*-test.

As expression of flagellar genes is directly controlled by FlhDC and FliA, we tested whether GmrA interacts with either or both. We found that *fliA* transcripts were significantly less abundant in the Δ*gmrA* mutant, a defect rescued by a low-copy plasmid carrying *gmrA* (**Figure 3A**). In contrast, expression of *flhD* and *flhC* was comparable among *E. coli* O157:H7 wild-type, Δ*gmrA*, and complemented strain (**Figure 3A**). Electrophoretic mobility shift and competition assays suggested that GmrA binds specifically to the *fliA* promoter *in vitro*, but not to the *flhDC* promoter and *rpoS* (negative control) (**Figure 3B**). ChIP-qPCR also showed that the *fliA* promoter was enriched 7.75-fold in GmrA-ChIP samples than in mock-ChIP control samples, confirming that GmrA binds to the *fliA* promoter *in vivo* (**Figure 3C**). However, *flhDC* promoter and *rpoS* were not enriched in GmrA-ChIP samples (**Figure 3C**). These results suggest that GmrA binds directly and specifically to the *fliA* promoter to upregulate transcription.

Motility and expression of *fliC* and *motA* were significantly reduced in the Δ*fliA* mutant compared with that in wild-type (**Figures 3D–F**), confirming that FliA is a positive regulator of bacterial motility and flagellar synthesis. We also found that the growth radius and abundance of *fliC* and *motA* transcripts were comparable between Δ*fliA* and the double mutant Δ*fliA*Δ*gmrA* (**Figures 3D–F**), *viz.*, deletion of *gmrA* has no impact on bacterial motility and flagellar gene expression in the Δ*fliA* background. In addition, both bacterial motility and flagellar gene expression were restored to wild-type levels when an inducible plasmid carrying *fliA* was introduced into the Δ*gmrA* mutant (**Figures 3D–F**). Collectively, the data indicate that GmrA regulates *E. coli* O157:H7 motility and flagella synthesis through *fliA*.

### Optimal Conditions for *gmrA* Expression Are Similar to Those in the Human Intestine

**FIGURE 4:**
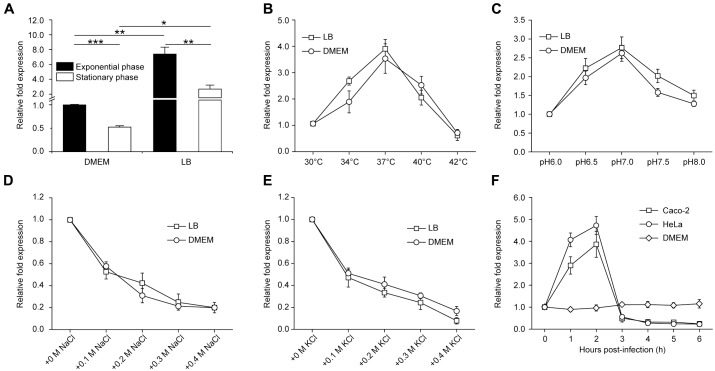
Optimal conditions for *gmrA* expression. **(A)** qRT-PCR for *gmrA* in exponential-phased and stationary-phased *E. coli* O157:H7 grown in LB or DMEM medium. qRT-PCR for *gmrA* in exponential-phased *E. coli* O157:H7 grown in LB or DMEM medium at different temperatures **(B)**, pH **(C)**, NaCl concentrations **(D)**, and KCl concentrations **(E)**. **(F)** qRT-PCR of *gmrA* in *E. coli* O157:H7 incubated in DMEM alone or co-incubated with Caco-2 or HeLa epithelial cells for 1 to 6 h. Data are mean ± SD, *n* = 3. ^∗^*P* ≤ 0.05; ^∗∗^*P* ≤ 0.01; ^∗∗∗^*P* ≤ 0.001 by Student’s *t*-test.

Expression of *gmrA* in *E. coli* O157:H7 grown to exponential phase or stationary phase in LB or DMEM (the tissue culture medium used for adherence assays) was examined by qRT-PCR. While the transcript level of *gmrA* was higher in LB-grown than the level in DMEM-grown *E. coli* O157:H7 under the same growth stage (**Figure 4A**), *gmrA* expressed higher in exponential phase than stationary phase *E. coli* O157:H7 grown in either medium (**Figure 4A**). To determine optimal conditions for *gmrA* expression, *E. coli* O157:H7 was grown in LB or DMEM at different temperatures, pH and osmolarity to exponential phase for qRT-PCR analysis. In either LB or DMEM, the optimal temperature for *gmrA* expression is 37°C, with transcript levels slightly reduced at 33 or 39°C, but significantly diminished at 30 or 42°C (**Figure 4B**). Buffering the medium at pH 7.0 elicited the highest levels of expression, while an increase or decrease in pH greatly reduced transcripts (**Figure 4C**). Expression was also maximal at physiological osmolarity, such that increased osmolarity from NaCl or KCl also significantly reduced transcription (**Figures 4D,E**). These results suggest that *gmrA* expression is maximal in conditions similar to those in the intestinal tract. However, *gmrA* expression was not affected by the presence of bile salts and sodium bicarbonate, which are predominantly found in small intestine and common signals for virulence gene regulation (Supplementary Figure S7). Remarkably, expression was strongly induced in the first 2 h of co-incubating *E. coli* O157:H7 with Caco-2 or HeLa epithelial cells, but was significantly repressed 3 h post-infection (**Figure 4F**), indicating that GmrA regulates motility and flagella synthesis mainly at the initial phase of infection. The expression of *gmrA* was at similar levels during the course of 6 h growth in DMEM in the absence of host epithelial cells (**Figure 4F**), indicating the induction of *gmrA* in the first 2 h and repression 3 h onwards is dependent on the presence of host cells.

### GmrA Is a Widespread Regulator of Bacterial Motility in Pathogenic *E. coli*

**FIGURE 5:**
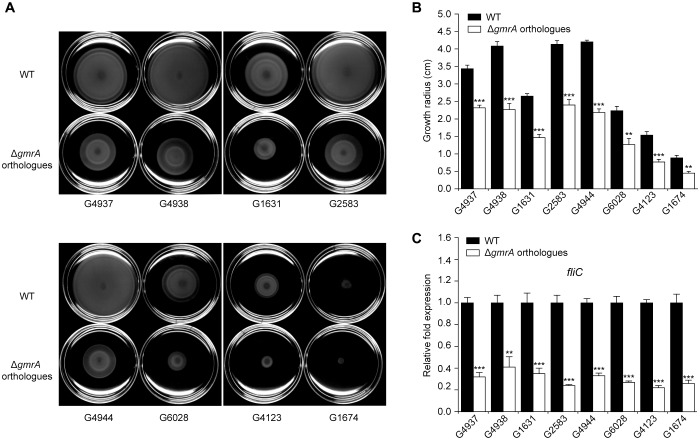
GmrA is a widespread regulator of bacterial motility and flagella synthesis. Representative images of swimming motility **(A)** and growth radius after 10 h at 30°C on motility agar **(B)** of various *E. coli* strains and corresponding mutants from which orthologous *gmrA* were deleted. **(C)** qRT-PCR for *fliC* in various strains grown to exponential phase, using 16S rRNA as internal control. **(B,C)** Data are mean ± SD, *n* = 3. ^∗∗^*P* ≤ 0.01; ^∗∗∗^*P* ≤ 0.001 by Student’s *t*-test.

Bioinformatics analysis of 231 available *E. coli* genome sequence showed that OI-29 and *gmrA* are highly conserved and widely distributed in various *E. coli* lineages. Phylogenetic analysis also revealed that *E. coli* with OI-29 fall predominantly into four distinct clades, consisting of all pathogenic strains with only one exception (64 in total) (Supplementary Figure S8 and Supplementary Table S4). Clade 1 contains enteropathogenic *E. coli* O55:H7 and EHEC strains O157:H7 and O145:H28. Other three clades contain neonatal meningitis-associated *E. coli* strains CE10, IHE3034, RS218, and S88; uropathogenic *E. coli* strains IAI39, MS6198, and PMV-1; extraintestinal pathogenic *E. coli* strains UTI89, UMN026, PCN033, and PPECC42; avian pathogenic *E. coli* strains O1 and IMT5155; enteroaggregative *E. coli* 042; adherent invasive *E. coli* UM146; and other clinical isolate *E. coli* strains (Supplementary Figure S8 and Supplementary Table S4). To investigate whether orthologous *gmrA* genes also regulate motility, we deleted such genes from eight representative strains, consisting of two strains each of O157:H7 and O55:H7 and one strain each of O127:H6, avian O2 and O2:H8, and neonatal meningitis-associated O18. The growth radius on motility plates and expression of *fliC* in these mutants were significantly decreased compared with the corresponding wild-type strains (**Figure 5**), confirming that GmrA is a widespread regulator of *E. coli* motility and flagellar synthesis.

## Discussion

In this study, we report that OI-29 is required for the motility of O157. This is the third motility associated OI identified in O157, indicating the importance of motility control for the evolving of this pathogen, through specifically acquired regulators. In contrast to previously reported OI-1 that encodes a *fliA* repressor (Z0021), and OI-172 that encodes the putative DEAH box RNA helicase (Z5898) to promote flagella-based motility via *fliC*, independent of *fliA* (Allison et al., 2012; Xu et al., 2013), OI-29 encoded GmrA promotes the *fliA* transcription, and thereby promotes flagellar synthesis. The regulation of *fliA* transcription by GmrA could be either direct or indirect, and this will be the subject of future studies. Our work significantly expands our understanding of bacterial motility control by providing a new example of *fliA*-dependent regulation, and increases the complexity of the regulatory network that governs flagellar genes. However, the two other OI-29 genes, *z0638* and *z0640*, have no obvious impact on O157:H7 motility, but may be involved in other bacterial processes, and thus require further characterization.

Bacterial flagellum is strictly regulated to prevent expression until environmental conditions are optimal, in order to avoid high metabolic cost and ensure survival in different environments (Allison et al., 2012). We found that transcription of *gmrA* was maximal at 37°C, pH 7.0, and physiological osmolarity, conditions that resemble those of the human intestinal tract. In addition, *gmrA* expression significantly increased in the first 2 h of co-incubating *E. coli* O157:H7 with Caco-2 intestinal cells, but diminished almost completely from 3 h onwards. Therefore, we appear to have identified a mechanism driving a previously reported phenomenon, in which flagella are liberally formed by *E. coli* O157:H7 in early stages of infection, but are subsequently lost (Mahajan et al., 2009). Accordingly, we propose that during early infection, *E. coli* O157:H7 upregulates *gmrA* in response to environmental changes in temperature, pH, osmolarity, and presence of host cells, ultimately activating the expression of flagellar genes through *fliA*. The resulting increase in motility then enables the pathogen to reach and adhere to colonization sites in the host. After successful infection, motility becomes less critical, and cells then downregulate *gmrA* to inhibit flagellar synthesis, not only to save energy, but also to minimize host immunity, since bacterial flagellin is a potent antigen that elicits secretion of proinflammatory chemokines in human intestinal epithelial cells (Berin et al., 2002; Miyamoto et al., 2006). Nevertheless, further studies are required to reveal the precise regulatory impact of GmrA in flagellar synthesis and pathogenesis *in vivo*. For example, how exactly GmrA-activated flagellar synthesis contributes to virulence, and whether other environmental cues in the human intestine elicit flagellar synthesis remains to be established, as are the mechanisms for regulating GmrA expression under host conditions.

The abilities to induce attaching and effacing lesions and to produce Shiga toxins are considered the two most important virulence determinants in *E. coli* O157:H7 (Monteiro et al., 2016). While GmrA has no effect on the expression of LEE genes that are responsible for forming such lesions, we further examined the expression of two representative Shiga toxin genes (*stx1A* and *stx2A*) in wild-type, ΔOI-29, and complemented strains. Results show that expression of both genes was not affected by deletion of OI-29 in *E. coli* O157:H7 (Supplementary Figure S9). Therefore, GmrA contributes to O157:H7 virulence by affecting motility while having no effects on lesion formation and Shiga toxin production.

OI-29 is widespread in different pathotypes of *E. coli* strains, and these strains cluster predominantly into four distinct clades. Hence, OI-29 was likely gained through four independent evolutionary events. For example, the most recent common ancestor of O55:H7, O157:H7, and O145:H28 in clade 1 seems to have acquired OI-29 after diverging from *E. coli* O157:H16. In addition, almost all strains with OI-29 are important pathogens in human or animals, indicating that these hosts may have driven the acquisition of OI-29 during the evolution of those pathogenic strains.

The motility and the expression of flagella genes (*fliA* and *fliC*) were also compared between *E. coli* O157:H7 and commensal *E. coli* K12. It was found that *E. coli* K12 was less motile on semi-solid LB agar than *E. coli* O157:H7 (Supplementary Figures S10A,B). In accordance, the transcriptional level of *fliA* and *fliC* was also lower in *E. coli* K12 (Supplementary Figure S10C). Both capacities of *E. col*i K12 were largely enhanced when an expression plasmid carrying *gmrA* was introduced into the strain (Supplementary Figure S10). These results suggest that *gmrA* was acquired by pathogenic *E. coli* strains during evolution from commensal strains to enhance motility, which may provide advantages in survival and infection *in vivo*.

## Conclusion

This study reveals a new example of regulators for the control of flagella synthesis. The laterally acquired GmrA is deployed by *E. coli* O157:H7 and likely many other pathogenic *E. coli* strains to enhance flagella synthesis and therefore motility during infection, highlighting the importance of motility in bacterial pathogenesis. The fact that GmrA is encoded in OI-29 demonstrates further that genomic islands contribute largely to bacterial pathogenesis by imparting new virulence traits, providing a rationale to investigate other uncharacterized OIs in *E. coli* O157:H7.

## Author Contributions

LF conceived and designed the experiments. BY, SW, JH, ZY, WH, LJ, and XL performed the experiments. BY and LF analyzed the data and wrote the paper.

## Conflict of Interest Statement

The authors declare that the research was conducted in the absence of any commercial or financial relationships that could be construed as a potential conflict of interest.
