# Supplementary material for: Transcriptional Activator GmrA, Encoded in Genomic Island OI-29, Controls the Motility of Enterohemorrhagic Escherichia coli O157:H7
Source: Front Microbiol. 2018 Feb 22;9:338. doi: 10.3389/fmicb.2018.00338 (PMC5826968; doi:10.3389/fmicb.2018.00338)
Supplement: Supplementary file 1 [file Table_1.DOCX]

Supplementary Material

Transcriptional Activator GmrA, Encoded in Genomic Island OI-29, Controls the Motility of Enterohemorrhagic *Escherichia coli* O157:H7

Bin Yang, Shaomeng Wang, Jianxiao Huang, Zhiqiu Yin, Lingyan Jiang, Wenqi Hou, Xiaomin Li, Lu Feng ^*^

*** Correspondence:** Lu Feng: fenglu63@nankai.edu.cn

# Supplementary Figures and Tables

## Supplementary Figures





**Supplementary Figure S1 |** **(A-B)** Representative images of swimming motility **(A)** and growth radius after 10 h at 30 °C on motility agar **(B)** of ΔOI-29, ΔOI-29 (pACYC184), Δ*gmrA*, and Δ*gmrA* (pACYC184). **(C-D)** Strains grown to exponential phase were analyzed by qRT-PCR for *fliA*, *fliC* and *motA*, using 16S rRNA as internal control. Data are mean ± s.d., n = 3.





**Supplementary Figure S2 |** **(A-B)** Representative images of swimming motility **(A)** and growth radius after 10 h at 37 °C on motility agar **(B)** of *E. coli* O157:H7 wild-type, ΔOI-29, ΔOI-29 (pACYC184), ΔOI-29 (pOI-29), Δ*gmrA*, Δ*gmrA* (pACYC184), and Δ*gmrA* (p*gmrA*). Data are mean ± s.d., n = 3. **, *P* ≤ 0.01; ***, *P* ≤ 0.001 by Student’s *t*-test.

**

**

**Supplementary Figure S3 |** **(A)** Graphical representation of OI-29. Island architecture and open reading frame nomenclature are based on the *E. coli* O157:H7 EDL933 sequence (GenBank: AE005174). **(B)** Schematic representation, sequence, location, and alignment of the DNA-binding domain in *gmrA*. The DNA-binding domain was predicted by NCBI conserved domain database (http://www.ncbi.nlm.nih.gov/Structure/cdd/wrpsb.cgi). Amino acids are numbered based on GmrA.

**

**

**Supplementary Figure S4 |** **(A-B)** Representative images of swimming motility **(A)** and growth radius after 10 h at 30 °C on motility agar **(B)** of *E. coli* O157:H7 wild-type, ΔOI-29 mutant, ΔOI-29 (p*z0638*) complemented strain, ΔOI-29 (p*gmrA*) complemented strain, and ΔOI-29 (p*z0640*) complemented strain. Data are mean ± s.d., n = 3. ***, *P* ≤ 0.001 by Student’s *t*-test.





**Supplementary Figure S5 |** **GmrA promotes *E. coli* O157:H7 motility. (A-D)** Representative images of swimming motility **(A)**, growth radius after 10 h at 30 °C on motility agar **(B)**, growth in LB medium **(C)**, and representative transmission electron micrographs **(D**; scale bar, 1 μm**)** of *E. coli* O157:H7 wild-type, Δ*gmrA* mutant, and complemented strain. The images of O157 WT in (**D**) is the same as that in Figure 2D. The average number of flagella per cell, as estimated from 500 cells per strain, is 1.21 for wild-type, 0.20 for Δ*gmrA* mutant, and 1.26 for the complemented strain. **(E)** Strains grown to exponential phase were analyzed by qRT-PCR for *fliC* and *motA*, using 16S rRNA as internal control. **(F)** Strains were also analyzed by immunoblotting for FliC, using DnaK as loading control. Bands were quantified by densitometry and normalized to DnaK. In (B), (E) and (F), data are mean ± s.d., n = 3. **, *P* ≤ 0.01; ***, *P* ≤ 0.001 by Student’s *t*-test.





**Supplementary Figure S6 |** **(A-B)** Representative images of swimming motility **(A)** and growth radius after 10 h at 30 °C on motility agar **(B)** of *E. coli* O157:H7 wild-type, Δ*z0638* mutant, and Δ*z0640* mutant. **(C)** Cells were also analyzed by qRT-PCR for *fliC* and *motA*. Data are mean ± s.d., n = 3.





**Supplementary Figure S7 |** **(A-B)** qRT-PCR for *gmrA* in exponential-phased *E. coli* O157:H7 grown in LB supplemented with different concentrations of bile salts **(A)** or sodium bicarbonate **(B)**. Data are mean ± s.d., n = 3.


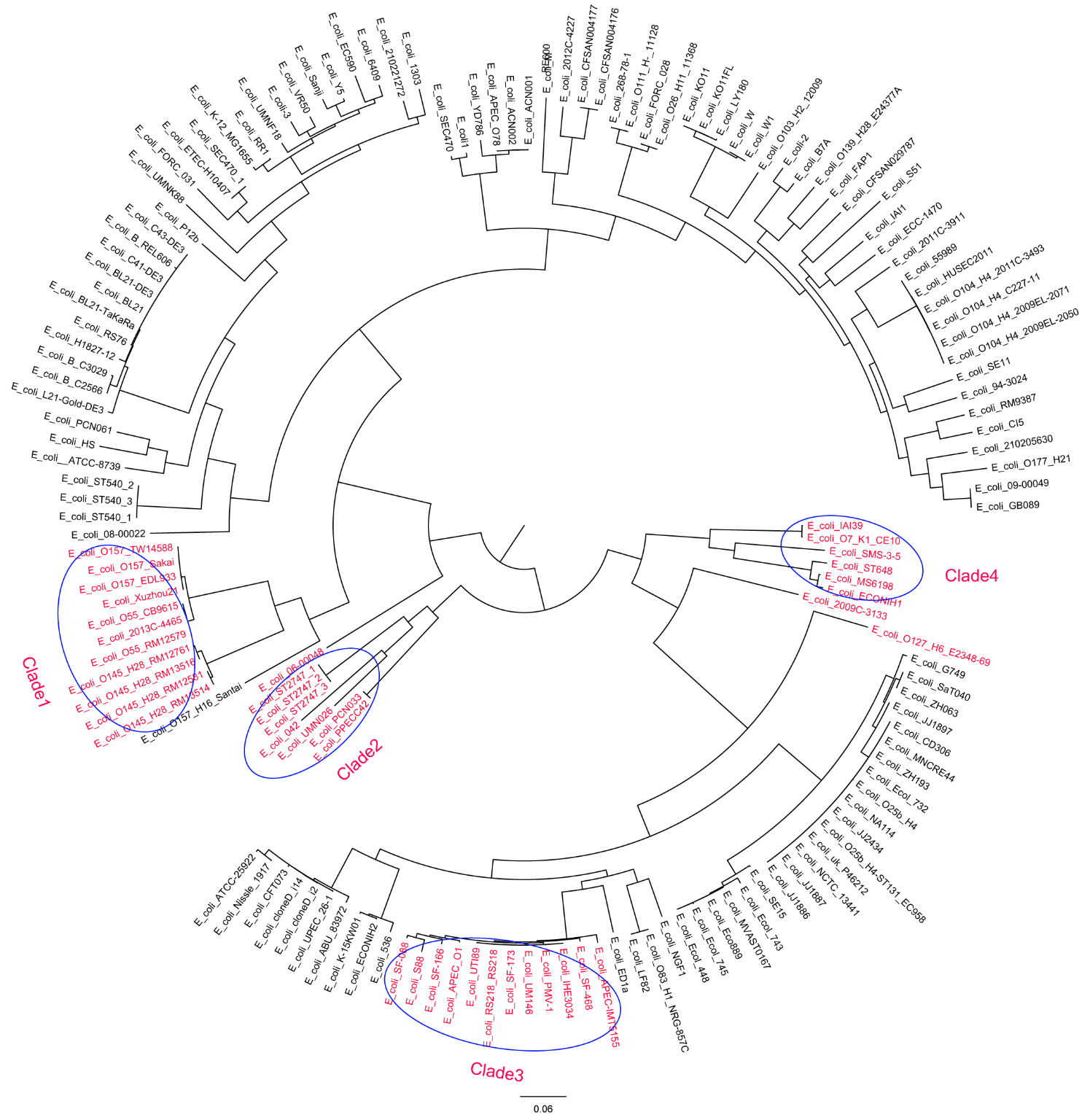


**Supplementary Figure S8 |** Maximum likelihood tree constructed in PhyML based on 1,406 single-copy core genes shared by 153 *E. coli* strains. Strains with OI-29 are indicated in red.





**Supplementary Figure S9 |** *E. coli* O157:H7 wild-type, ΔOI-29 mutant, and ΔOI-29 complemented strain were grown to exponential phase, and analyzed by qRT-PCR for the expression of Shiga toxin genes, using 16S rRNA as internal control. Data are mean ± s.d., n = 3.





**Supplementary Figure S10 |** Representative images of swimming motility **(A)** and growth radius after 10 h at 30 °C on motility agar **(B)** of *E. coli* O157:H7, *E. coli* K12, and *E. coli* K12 (p*gmrA*). **(C)** Cells were also analyzed by qRT-PCR for *fliA* and *fliC*. Data are mean ± s.d., n = 3. *, *P* ≤ 0.05; **, *P* ≤ 0.01; ***, *P* ≤ 0.001 by Student’s *t*-test.


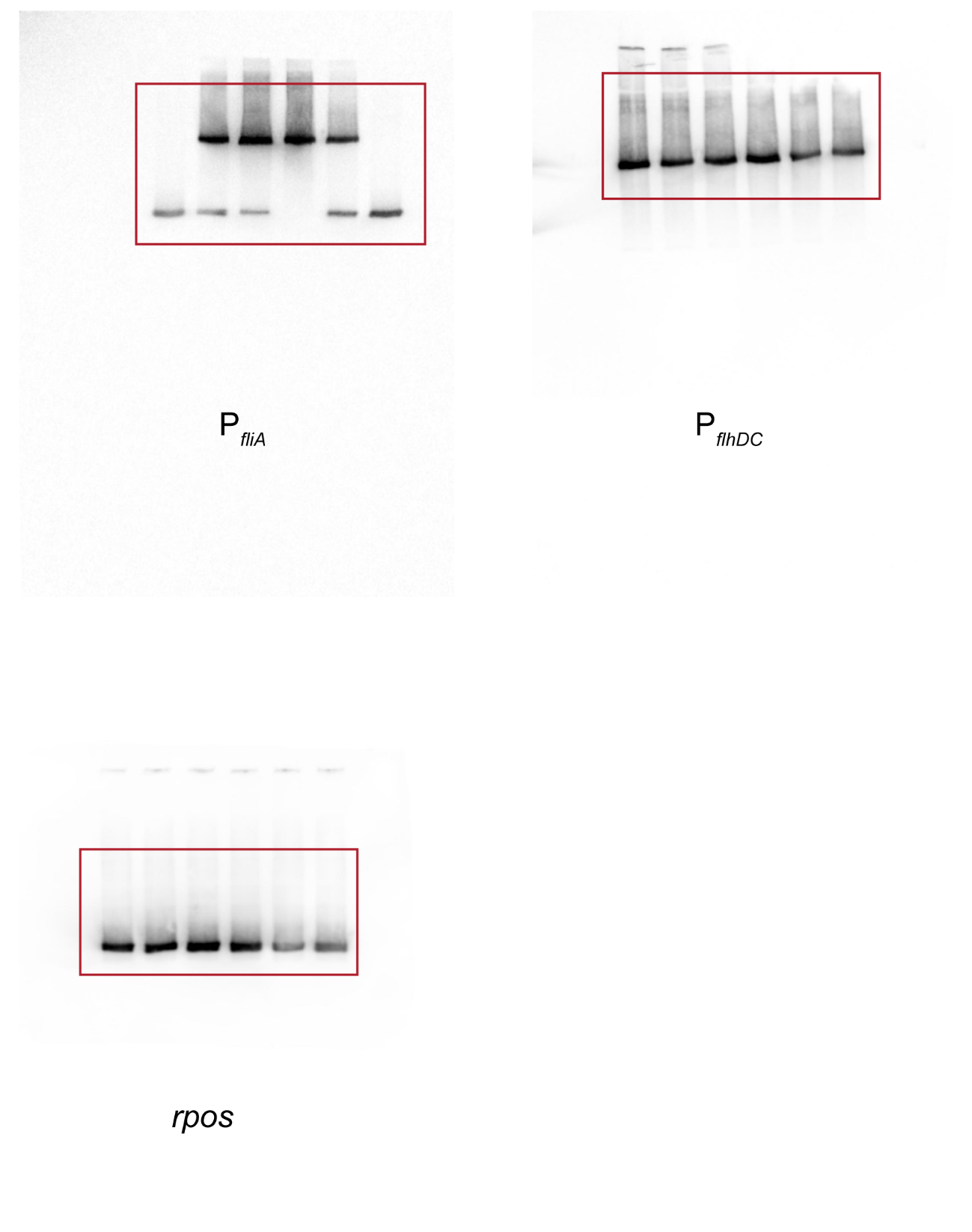


**Supplementary Figure S11 |** Full blots of the EMSAs. Boxes highlight lanes used in the Figure 3B.

## Supplementary Tables

**Supplementary Table S1.** Strains and plasmids used in this study

| Strains | Genotype or description | Source or reference |
| --- | --- | --- |
| G2734 | Wild-type EHEC O157:H7 EDL933 | ATCC^*^ |
| H2719 | OI-29 deletion mutant in G2734 | This work |
| H2720 | *z0638* deletion mutant in G2734 | This work |
| H2721 | *gmrA* deletion mutant in G2734 | This work |
| H2722 | *z0640* deletion mutant in G2734 | This work |
| H2723 | *fliA* deletion mutant in G2734 | This work |
| H2724 | *fliA* and *gmrA* double deletion mutant in G2734 | This work |
| G4908 | Wild-type *E. coli* K12 | Aus^†^ |
| G4937 | Wild-type EHEC O157:H7 (*eae*+, *stx*1+, *stx*2+) | ([Kurioka et al., 1998](#_ENREF_5)) |
| H2725 | *gmrA* orthologues deletion mutant in G4937 | This work |
| G4938 | Wild-type EHEC O157:H7 (*eae*+, *stx*2+) | ([Gansheroff et al., 1999](#_ENREF_4)) |
| H2726 | *gmrA* orthologues deletion mutant in G4938 | This work |
| G1631 | Wild-type APEC O2:H8 | Aus |
| H2727 | *gmrA* orthologues deletion mutant in G1631 | This work |
| G2583 | Wild-type EPEC O55:H7 CB9615 | BfR^‡^ |
| H2728 | *gmrA*orthologues deletion mutant in G2583 | This work |
| G4944 | Wild-type EPEC O55:H7 | Aus |
| H2729 | *gmrA* orthologues deletion mutant in G4944 | This work |
| G6028 | Wild-type EPEC O127:H6 E2348/69 | FDU^§^ |
| H2730 | *gmrA* orthologues deletion mutant in G6082 | This work |
| G4123 | Wild-type NMEC O18:K1:H7 RS218 | JHU ^¶^ |
| H2731 | *gmrA* orthologues deletion mutant in G4123 | This work |
| G1674 | Wild-type APEC O2 | Aus |
| H2732 | *gmrA* orthologues deletion mutant in G1674 | This work |
| H2733 | H2719 containing pLW1847 | This work |
| H2734 | H2719 containing pLW1848 | This work |
| H2735 | H2719 containing pLW1849 | This work |
| H2736 | H2719 containing pLW1850 | This work |
| H2737 | H2721 containing pLW1849 | This work |
| H2738 | BL21 containing pLW1851 | This work |
| H2739 | H2721 containing pLW1852 | This work |
| H2740 | H2721 containing pLW1853 | This work |
| Plasmids | | |
| pKD46 | Red recombination plasmid, ApR | ([Datsenko and Wanner, 2000](#_ENREF_3)) |
| pKD3 | Containing a chloramphenicol resistance cassette and the flipase recognition sites, CmR | ([Datsenko and Wanner, 2000](#_ENREF_3)) |
| pKD4 | Containing a kanamycin resistance cassette and the flipase recognition sites, KmR | ([Datsenko and Wanner, 2000](#_ENREF_3)) |
| pCP20 | FLP expression plasmid, ApR, CmR | ([Datsenko and Wanner, 2000](#_ENREF_3)) |
| pACYC184 | Low copy expression vector, CmR, TcR | ([Chang and Cohen, 1978](#_ENREF_2)) |
| pTRC99A | Expression vector, ApR | ([Amann et al., 1988](#_ENREF_1)) |
| pET-28a | T7 expression vector, KmR | Novagen |
| pLW1847 | pACYC184 carrying OI-29, CmR | This work |
| pLW1848 | pACYC184 carrying *z0638*, CmR | This work |
| pLW1849 | pACYC184 carrying *gmrA*, CmR | This work |
| pLW1850 | pACYC184 carrying *z0640*, CmR | This work |
| pLW1851 | pET-28a carrying *gmrA*, KmR | This work |
| pLW1852 | pTRC99A carrying *gmrA*-3×FLAG, ApR | This work |
| pLW1853 | pACYC184 carrying *fliA*, CmR | This work |

^*^, ATCC, American Type Culture Collection, Manassas, Virginia, USA. ^†^, Aus, School of Molecular and Microbial Biosciences, University of Sydney, Sydney, Australia. ^‡^, BfR, Beutin Federal institute for Risk Assessment National Reference Laboratory for *Escherichia coli,* Berlin, Germany. ^§^, FDU, Department of Immunology, School of Basic Medical Sciences, Fudan University, Shanghai, China. ^¶^, JHU, Division of Pediatric Infectious Diseases, Johns Hopkins University School of Medicine.

**Supplementary Table S2.** Primers used in this study (5'-3')

| Primers for gene mutation | | |
| --- | --- | --- |
| OI-29 | F | AAAATCTCTCCGGCTGCTGTCATCATCGGGCGGGGTGAGTGTAGGCTGGAGCTGCTTCG |
| OI-29 | R | ATTGCGATAGAAGGGATAACGCTGATCATCCGTGCGGTCCATATGAATATCCTCCTTAG |
| *z0638* | F | AATCATAATATGCGTCAGGGGTATTTTCCTTTGCTCCTGTGTAGGCTGGAGCTGCTTCG |
| *z0638* | R | TCACCTCTTCATTAATTTGTACATTATTTAAGGTTAACAATGGGAATTAGCCATGGTCC |
| *gmrA* | F | AAGCTTCCTTGCTCTTATTTATATTGAGGATTGAAATAGTGTAGGCTGGAGCTGCTTCG |
| *gmrA* | R | GAAACATTAATACGACTTATTTTGAACAAGAGAAAATGAATGGGAATTAGCCATGGTCC |
| *z0640* | F | CAATTTTTAAGAGGTTTACGCCAGGCAGGAAAATTTAAGTGTAGGCTGGAGCTGCTTCG |
| *z0640* | R | CCGCCTCCGCCATGTTAAATGTTAACTGGCATTGGCAATATGGGAATTAGCCATGGTCC |
| *fliA* | F | TCCTGGTAGTCAAAGTTAAAGTGCGGCATTTACTGACGGTGTAGGCTGGAGCTGCTTCG |
| *fliA* | R | AATCATGCCGATAACTCATATAACGCAGGGCTGTTTATCATGGGAATTAGCCATGGTCC |
| *escC* | F | CCAACACACTTGTTTTCTGATATAGGACGAATTGTGTAGTGTAGGCTGGAGCTGCTTCG |
| *escC* | R | TCTAGAGAGCTAAATTTCCTGCTCATAAAGCCTCATTTACATATGAATATCCTCCTTAG |
| *gmrA-*01^*^ | F | AAGCTTCCTTGCTCTTATTTATATTGAGGATTGAAATAGTGTAGGCTGGAGCTGCTTCG |
| *gmrA-*01 | R | GAAACATTAATACGACTTATTTTGAACAAGAGAAAATGAATGGGAATTAGCCATGGTCC |
| *gmrA-*02^†^ | F | AAGCTTCCTTGCTCTTATTTATATTGAGGATTGAAATAGTGTAGGCTGGAGCTGCTTCG |
| *gmrA-*02 | R | GAAACATCAATGCGACTTATTTTGAACAAGAGAAAATGAATGGGAATTAGCCATGGTCC |
| *gmrA-*03^‡^ | F | ATGTGCGGAAAATTGCGAATAAGAAATTGCTGTTAAAAGTGTAGGCTGGAGCTGCTTCG |
| *gmrA-*03 | R | GAAACATTAATGCAACTTATTTTGATCGAGAGAAAATGAATGGGAATTAGCCATGGTCC |
| *gmrA-*04^§^ | F | AAGCTTCCTTGCTCTTATTTATATTGAGGATTGAAATAGTGTAGGCTGGAGCTGCTTCG |
| *gmrA-*04 | R | GAAACATTAATGCGACTTATTTTGAACAAGAGAAAATGAATGGGAATTAGCCATGGTCC |
| Primers for mutant verification | | |
| OI-29 | F | CTACACGCAGCAGCATCTCA |
| OI-29 | R | GCTGTGGTGGAAATGGTTGT |
| *z0638* | F | TGAACCAGGGAGGCTAATAA |
| *z0638* | R | CTGCGGTGTCTGTCGGA |
| *gmrA* | F | GCGGGAAGTGGGTGAA |
| *gmrA* | R | CAAATGGCGGCGTAGAT |
| *z0640* | F | ATTTCGTGCTCTTTCAATGT |
| *z0640* | R | TGCTGGAATCTCCGCTG |
| *fliA* | F | CAATGTAAATCACCGCAAA |
| *fliA* | R | ACCCCCAAATAACCCCT |
| *escC* | F | TCTGCAATATCCTGGCCGTT |
| *escC* | R | GCTAATGTGCCACCATCCTG |
| *gmrA-*05^¶^ | F | GCGGGAAGTGGGTGAA |
| *gmrA-*05 | R | CAAATGGCGGCGTAGAT |
| *gmrA-*04 | F | GTAATATAACCGCCAGCAGG |
| *gmrA-*04 | R | TAGCACACATCAGGAACCG |
| Primers for gene cloning | | |
| OI-29 | F | GCTCTAGAAAAATCAGTGAATCCCCGT |
| OI-29 | R | CGGGATCCATCATCCGTGCGGTCATC |
| *z0638* | F | GCTCTAGACGTAAAGAAGCCAATGCG |
| *z0638* | R | CGGGATCCGCGTCAGGGGTATTTTCC |
| *gmrA* | F-1 | GCTCTAGAAAAGAAACGCATTTTAGCAC |
| *gmrA* | R-1 | CGGGATCCATTAGAACGAATATCCCACG |
| *gmrA* | F-2 | CGGGATCCATGAAAACTGTAAACGTAGCTTTA |
| *gmrA* | R-2 | CCGCTCGAGTGAGGATTGAAATATTAGAACG |
| *z0640* | F | GCTCTAGATTCATTCATTTTCTCTTGTTCA |
| *z0640* | R | CGGGATCCGTCATATCACGCCAAAACC |
| *fliA* | F | GCTCTAGACGCAGCAGGTTCTGTCTC |
| *fliA* | R | CGGGATCCGCCGTCTTTTCAGGTGCT |
| Primers for *gmrA*-3×FLAG | | |
| *gmrA*-3F | F | CATGCCATGGGAATGAAAACTGTAAACGTAGCTTTA |
| *gmrA*-3F | R | CGGGATCCTTACTATTTATCGTCGTCATCTTTGTAGTCGATATCATGATCTTTATAATCACCGTCATGGTCTTTGTAGTCGAACGAATATCCCACGCCG |
| Primers for qRT-PCR | | |
| *rrsH* | F | GAAAGCGTGGGGAGCAAAC |
| *rrsH* | R | ACATGCTCCACCGCTTGTG |
| *eae* | F | GACGGTAGTTCACTGGACTTCTT |
| *eae* | R | TCGCCACCAATACCTAAACG |
| *tir* | F | AAAGCAGCAGGCGAAGAGG |
| *tir* | R | TCGGCACCTGCGAATCAT |
| *ler* | F | CAGGAAGCAAAGCGACTG |
| *ler* | R | ACCAGGTCTGCCCTTCTT |
| *escT* | F | GCAATAGATGCGGCTGGAC |
| *escT* | R | TCGGCTTGTAATGGTAATATCTCG |
| *escC* | F | GACCAAAATGTTGTCGTCCCA |
| *escC* | R | AGGTTACCGCTTCGCTCG |
| *escN* | F | AGGTTTTCTTGTTGCCTTTTGA |
| *escN* | R | TCTCCATTGGTCTGCCTATGC |
| *espB* | F | AAAACTCCTCGGCAAGATGG |
| *espB* | R | AATAATCCCGCCAACCAAAG |
| *z0638* | F | GTAGTTCACCCACTTCCCGC |
| *z0638* | R | TGCTCATTCTAAAGGTCATGCC |
| *gmrA* | F | CATCGCTATCGCCTCACAAC |
| *gmrA* | R | CCACGCCGACACCTGC |
| *z0640* | F | GCATTGCTACCGATCCTTTTTC |
| *z0640* | R | CCTTTACTCATACTTTTTTGGTCTTC |
| *fliC* | F | ACAACGCTGGTAGCGCAGCT |
| *fliC* | R | GGCAGCCGCTTTGGTTTCGC |
| *motA* | F | GCGAACAGTCTGGCGCTGGT |
| *motA* | R | TGTGCGATAAGCGCCCCCAG |
| *fliA* | F | GGATAAACACTCGCTGTGGCAG |
| *fliA* | R | GAAGTTCATCCAGCATAGCGCC |
| *flhD* | F | ACCTCCGAGTTGCTGAAACAC |
| *flhD* | R | TTGCTGGAGATCGTCAACGC |
| *flhC* | F | CCCGCAAGCAGAAGAAGGA |
| *flhC* | R | GCTGGTGAGCGTGGGTAATAA |
| *stx1A* | F | TGGATGATCTCAGTGGGCGT |
| *stx1A* | R | GCCACGCTTCCCAGAATTGC |
| *stx2A* | F | GCACTGTCTGAAACTGCTCCTG |
| *stx2A* | R | ATAACGGCCACAGTCCCCAG |
| Primers for EMSA | | |
| P*_fliA_* | F | CTGCCACAGCGAGTGTTT |
| P*_fliA_* | R | CCGACTATGCGACCGAA |
| P*_flhDC_* | F | TTCCCACCCAGAATAACCA |
| P*_flhDC_* | R | TTGATTGTTGCCTTTCTTTGT |
| *rpoS* | F | CTTCCAGTGTTGCCGCT |
| *rpoS* | R | CCCGTACTATTCGTTTGCC |
| Primers for ChIP-qPCR | | |
| P*_fliA_* | F | CAGGTAAATTCCAGGCAGAAAA |
| P*_fliA_* | R | CTGAGACTGACGGCAACGC |
| P*_flhDC_* | F | CACTACACGCACATACAACGGA |
| P*_flhDC_* | R | GATTGAAATACACCCAAAACAAAAG |
| *rpoS* | F | GTTATCGCAGGGAGCCACA |
| *rpoS* | R | TTTTACCACCAGACGCAAGTTA |

^*^, *gmrA-*01, orthologous *gmrA* genes in G4937, G4938, G2583 and G4944; ^†^, *gmrA-*02, orthologous *gmrA* gene in G4123; ^‡^, *gmrA-*03, orthologous *gmrA* gene in G6082; ^§^, *gmrA-*04, orthologous *gmrA* genes in G1631 and G1674; ^¶^, *gmrA-*05, orthologous *gmrA* genes in G4937, G4938, G2583, G4944, G4123 and G6082.

**Supplementary Table S3.** Relative fold changes in the expression of OI-29 genes in EHEC O157:H7 after 3 h of incubation with HeLa cells

| Gene | Product/Function | RPKM  DMEM-grown O157 | RPKM  HeLa-attached O157 | Fold^*^ | *P* value | Fold^†^ |
| --- | --- | --- | --- | --- | --- | --- |
| *z0638* | hypothetical protein | 20.38 | 0.00 | 0.00 | 2.18E-04 | -10.63±2.25 |
| *gmrA* | hypothetical protein | 96.11 | 0.00 | 0.00 | 1.23E-09 | -8.48±1.45 |
| *z0640* | hypothetical protein | 43.52 | 0.00 | 0.00 | 8.76E-07 | -5.19±1.37 |

^*^, Fold change measured via comparative transcriptome analysis; ^†^, Fold change measured via qRT-PCR, the presented values are the mean±SD of three independent experiments; -, down-regulated; *P* values were calculated using binomial test.

**Supplementary Table S4.** The prevalence of OI-29 among different *E. coli* strains

| Strains | Clade | Pathotype or Origin |
| --- | --- | --- |
| *E. coli* NADC 5670 6564 | 1 | Enterohemorrhagic *E. coli* |
| *E. coli* NADC 5670 6565 | 1 | Enterohemorrhagic *E. coli* |
| *E. coli* O157 WS4202 | 1 | Enterohemorrhagic *E. coli* |
| *E. coli* O157 ELD 933-1 | 1 | Enterohemorrhagic *E. coli* |
| *E. coli* O157 ELD 933 | 1 | Enterohemorrhagic *E. coli* |
| *E. coli* O157 Sakai | 1 | Enterohemorrhagic *E. coli* |
| *E. coli* SRCC 1675 | 1 | Enterohemorrhagic *E. coli* |
| *E. coli* O157 3384 | 1 | Enterohemorrhagic *E. coli* |
| *E. coli* O157 TW14588 | 1 | Enterohemorrhagic *E. coli* |
| *E. coli* PA20 | 1 | Enterohemorrhagic *E. coli* |
| *E. coli* O157 8368 | 1 | Enterohemorrhagic *E. coli* |
| *E. coli* Xuzhou21 | 1 | Enterohemorrhagic *E. coli* |
| *E. coli* O157 SS52 | 1 | Enterohemorrhagic *E. coli* |
| *E. coli* O157 SS17 | 1 | Enterohemorrhagic *E. coli* |
| *E. coli* O157 JEONG-1266 | 1 | Enterohemorrhagic *E. coli* |
| *E. coli* O157 TW14359 | 1 | Enterohemorrhagic *E. coli* |
| *E. coli* O157 EC4115 | 1 | Enterohemorrhagic *E. coli* |
| *E. coli* 28RC1 | 1 | Enterohemorrhagic *E. coli* |
| *E. coli* O157 FRIK2533 | 1 | Enterohemorrhagic *E. coli* |
| *E. coli* O157 FRIK2069 | 1 | Enterohemorrhagic *E. coli* |
| *E. coli* O157 FRIK2455 | 1 | Enterohemorrhagic *E. coli* |
| *E. coli* O157 FRIK944 | 1 | Enterohemorrhagic *E. coli* |
| *E. coli* O157 664-PT8 | 1 | Enterohemorrhagic *E. coli* |
| *E. coli* O157 180-PT54 | 1 | Enterohemorrhagic *E. coli* |
| *E. coli* O157 2159 | 1 | Enterohemorrhagic *E. coli* |
| *E. coli* O157 2149 | 1 | Enterohemorrhagic *E. coli* |
| *E. coli* O157 1130 | 1 | Enterohemorrhagic *E. coli* |
| *E. coli* O157 9234 | 1 | Enterohemorrhagic *E. coli* |
| *E. coli* O157 4276 | 1 | Enterohemorrhagic *E. coli* |
| *E. coli* O55 CB9615 | 1 | Enteropathogenic *E. coli* |
| *E. coli* 2013C-4465 | 1 | Enteropathogenic *E. coli* |
| *E. coli* O55 RM12579 | 1 | Enteropathogenic *E. coli* |
| *E. coli* O145:H28 RM12581 | 1 | Enterohemorrhagic *E. coli* |
| *E. coli* O145:H28 RM13514 | 1 | Enterohemorrhagic *E. coli* |
| *E. coli* O145:H28 RM12761 | 1 | Enterohemorrhagic *E. coli* |
| *E. coli* O145:H28 RM13516 | 1 | Enterohemorrhagic *E. coli* |
| *E. coli* 06-00048 |  | Foodborne pathogen |
| *E. coli* O7:K1 CE10 | 2 | Neonatal-meningitis-associated *E. coli* |
| *E. coli* IAI39 | 2 | Uropathogenic *E. coli* |
| *E. coli* ECONIH1 | 2 | hospital-associated carbapenemase-producing *Enterobacteriaceae* |
| *E. coli* MS6198 | 2 | Uropathogenic *E. coli* |
| *E. coli* ST648 | 2 | Clinical isolate *E. coli* from pleural effusion of patients with empyema thoracis |
| *E. coli* SMS-3-5 | 2 | Environmental isolate *E. coli* |
| *E. coli* APEC-IMT5155 | 3 | Avian pathogenic *E. coli* |
| *E. coli* UTI89 | 3 | Extraintestinal pathogenic *E. coli* |
| *E. coli* RS218 | 3 | Neonatal-meningitis-associated *E. coli* |
| *E. coli* PMV-1 | 3 | Uropathogenic *E. coli* |
| *E. coli* UM146 | 3 | Enteroinvasive *E. coli* |
| *E. coli* SF-173 | 3 | Clinical isolate *E. coli* from bloodstream infections |
| *E. coli* IHE3034 | 3 | Neonatal-meningitis-associated *E. coli* |
| *E. coli* SF-088 | 3 | Clinical isolate *E. coli* from bloodstream infections |
| *E. coli* S88 | 3 | Neonatal-meningitis-associated *E. coli* |
| *E. coli* SF-166 | 3 | Clinical isolate *E. coli* from bloodstream infections |
| *E. coli* APEC O1 | 3 | Avian pathogenic *E. coli* |
| *E. coli* SF-468 | 3 | Clinical isolate *E. coli* from bloodstream infections |
| *E. coli* O127:H6 E2348-69 |  | Enterohemorrhagic *E. coli* |
| *E. coli* 2009C-3133 |  | Shiga toxin-producing *E. coli* |
| *E. coli* 042 | 4 | Enteroaggregative *E. coli* |
| *E. coli* UMN026 | 4 | Extraintestinal pathogenic *E. coli* |
| *E. coli* ST2747-1 | 4 | Clinical isolate *E. coli* from stool samples from two Belgian outpatients with urinary tract infections |
| *E. coli* ST2747-2 | 4 | Clinical isolate *E. coli* from stool samples from two Belgian outpatients with urinary tract infections |
| *E. coli* ST2747-3 | 4 | Clinical isolate *E. coli* from stool samples from two Belgian outpatients with urinary tract infections |
| *E. coli* PCN033 | 4 | Extraintestinal pathogenic *E. coli* |
| *E. coli* PPECC42 | 4 | Extraintestinal pathogenic *E. coli* |

# Supplementary References

Amann, E., Ochs, B., and Abel, K.J. (1988). Tightly regulated *tac* promoter vectors useful for the expression of unfused and fused proteins in *Escherichia coli*. *Gene*. 69**,** 301-315.

Chang, A.C., and Cohen, S.N. (1978). Construction and characterization of amplifiable multicopy DNA cloning vehicles derived from the P15A cryptic miniplasmid. *J Bacteriol*. 134**,** 1141-1156.

Datsenko, K.A., and Wanner, B.L. (2000). One-step inactivation of chromosomal genes in *Escherichia coli* K-12 using PCR products. *Proc Natl Acad Sci U S A*. 97**,** 6640-6645. doi: 10.1073/pnas.120163297.

Gansheroff, L.J., Wachtel, M.R., and O'Brien, A.D. (1999). Decreased adherence of enterohemorrhagic *Escherichia coli* to HEp-2 cells in the presence of antibodies that recognize the C-terminal region of intimin. *Infect Immun*. 67**,** 6409-6417.

Kurioka, T., Yunou, Y., and Kita, E. (1998). Enhancement of susceptibility to Shiga toxin-producing *Escherichia coli* O157:H7 by protein calorie malnutrition in mice. *Infect Immun*. 66**,** 1726-1734.
